# Targeted Proteomics Enables Simultaneous Quantification of Folate Receptor Isoforms and Potential Isoform-based Diagnosis in Breast Cancer

**DOI:** 10.1038/srep16733

**Published:** 2015-11-17

**Authors:** Ting Yang, Feifei Xu, Danjun Fang, Yun Chen

**Affiliations:** 1 Department of Pharmacy, Nanjing Drum Tower Hospital, The Affiliated Hospital of Nanjing University Medical School, Nanjing, 210029, China; 2 School of Pharmacy, Nanjing Medical University, Nanjing, 211166, China

## Abstract

The distinct roles of protein isoforms in cancer are becoming increasingly evident. FRα and FRβ, two major isoforms of the folate receptor family, generally have different cellular distribution and tissue specificity. However, the presence of FRβ in breast tumors, where FRα is normally expressed, complicates this situation. Prior to applying any FR isoform-based diagnosis and therapeutics, it is essential to monitor the expression profile of FR isoforms in a more accurate manner. An LC-MS/MS-based targeted proteomics assay was developed and validated in this study because of the lack of suitable methodology for the simultaneous and specific measurement of highly homologous isoforms occurring at low concentrations. FRα and FRβ monitoring was achieved by measuring their surrogate isoform-specific peptides. Five human breast cell lines, isolated macrophages and 60 matched pairs of breast tissue samples were subjected to the analysis. The results indicated that FRβ was overexpressed in tumor-associated macrophages (TAMs) but not epithelial cells, in addition to an enhanced level of FRα in breast cancer cells and tissue samples. Moreover, the levels of the FR isoforms were evaluated according to the histology, histopathological features and molecular subtypes of breast cancer. Several positive associations with PR/ER and HER2 status and metastasis were revealed.

The clinical relevance of specific protein isoforms is becoming increasingly clear, particularly in cancer diagnosis and treatment. However, the ability to discover, validate and utilize protein isoforms in clinical practice is severely limited by the existing laboratory tools. Among these attempts, the investigation of the folate receptor (FR) family is facing a more complex situation. This protein family can mediate uni-directional folate transport into cells and is one of the most potent targets in cancer^1^. Among the four identified isoforms of FR (α, β, γ, and δ), FRα and FRβ are 38 kDa membrane-associated proteins that have a high affinity for folate^2^. In addition, they are both active when the folate supply is low or when rapid cell growth requires elevated folate uptake. Despite these similarities and the known high frequency in cancers, the two isoforms have different cellular distribution patterns and tissue specificity^3^,^4^. In general, FRα is expressed in certain normal epithelial cells, and its expression is markedly enhanced in several carcinomas, including breast cancer, whereas FRβ is often overexpressed in non-epithelial malignancies^4^. Thus, FRα and FRβ are not expected to be observed together. However, several recent reports have shown the presence of FRβ as well as FRα in breast tumors^5^. The subsequent investigation suggested that the detected FRβ was actually not present on the tumor itself, but on tumor-associated macrophages (TAMs) that accumulated at the tumor sites^5^.

As a leading cause of death in women, breast cancer is highly complex, with cancer cells constituting only one of many distinct cell types. Indeed, within breast tumors, cancer cells represent only a small proportion (<20%) of the total cell number^6^. The remaining cell types, including TAMs, are often grouped together under the collective term of “tumor-associated stroma”. The appearance of FRβ in TAMs suggested that FRβ may be a useful breast cancer marker and a potential target for anti-cancer therapy, in addition to FRα, because breast cancer development and progression are highly dependent on specialized tumor-associated stroma^7^, as tumors rarely develop in the absence of this microenvironment. However, the expression profile of FR isoforms across the disease is unknown, making it difficult to apply FR isoform-based diagnosis and therapeutics. It is essential to monitor the level of FR isoforms in a more accurate manner to comprehensively assess their clinical significance.

To date, numerous reports have demonstrated that protein isoform distributions are related to clinical effects. However, there is a lack of suitable methods for the simultaneous and specific measurement of highly homologous isoforms occurring at low concentrations in biological samples. Antibody-based techniques, including Western blotting^8^ and immunohistochemistry (IHC)^9^, have been traditionally employed to measure protein levels. Using these techniques, the detection of protein isoforms is mainly based on a panel of antibodies that recognize distinct epitopes and different electrophoresis motilities^10^. While these techniques provide valuable information on protein levels, they remain to be improved due to the lack of the necessary specificity and reproducibility^11^. In addition, one isoform is usually targeted at a time because the methods for developing multiple colors are complicated due to fluorescence spectral overlap^12^. Furthermore, it should be noted that one can compare the expression levels of each isoform only when the antibody recognizes an identical epitope per isoform^10^. More importantly, most of the assays that have been developed are qualitative or semi-quantitative but not quantitative^13^.

Liquid chromatography-tandem mass spectrometry (LC-MS/MS)-based targeted proteomics may represent a promising alternative method for protein isoform quantification. In a targeted protein analysis, selected/multiple reaction monitoring (SRM or MRM) on a triple quadrupole instrument is generally employed, and LC-MS/MS assays are developed to detect fragment ion signals from proteolytic peptides representing the targeted protein^14^,^15^. The precursor/product ion *m/z* pair, referred to as the transition, is used to yield the chromatogram. The area under the chromatogram curve provides a quantitative measurement for each desired peptide and target protein. Thus, simultaneous monitoring of protein isoforms may be achieved by measuring the surrogate peptides that are specific to each isoform^16^. Indeed, a similar approach has been successfully used to monitor several protein isoform occurrences^16^,^17^,^18^.

This study first developed and validated an LC-MS/MS-based targeted proteomics assay for the simultaneous quantification of FRα and FRβ in human breast cells and tissue samples. This assay was then applied to the quantitative analysis of FR isoforms in the MCF-10 A normal breast cell line; the MCF-7/WT, T47D, MDA-MB-231 and MCF-7/ADR breast cancer cell lines; macrophages isolated from fresh breast tissue; and 60 pairs of primary human breast tumors and adjacent normal tissue samples. The resulting values were compared to those obtained using Western blotting and IHC. Finally, the biological distribution of the FRs and their differential levels based on breast cancer histology, clinical histopathological features and molecular subtypes were also discussed.

## Materials and Methods

### Chemicals and Reagents

Stable isotope-labeled amino acids were supplied by Cambridge Isotope Laboratories, Inc. (Andover, MA, USA). The synthetic proteolytic peptides and their corresponding stable isotope-labeled internal standards were synthesized by ChinaPeptides Co., Ltd. (Shanghai, China). FRα and FRβ were purchased from Abnova (Taipei, Taiwan) and Abcam (Cambridge, UK), respectively. Ammonium bicarbonate (NH_4_HCO_3_) was obtained from Qiangshun Chemical Reagent Co., Ltd. (Shanghai, China). DL-dithiothreitol (DTT), iodoacetamide (IAA), Tris-HCl and Triton X-114 were purchased from Sigma-Aldrich (St. Louis, MO, USA). Ethylenediaminetetraacetic acid disodium salt (EDTA-2Na) was obtained from Sinopharm Chemical Reagent Company (Shanghai, China). Sequencing grade modified trypsin was purchased from Promega (Madison, WI, USA) Phosphate buffered saline (PBS) was purchased from the Beyotime Institute of Biotechnology (Jiangsu, China). Acetonitrile (ACN) and methanol were obtained from Tedia Company, Inc. (Fairfield, OH, USA). Trifluoroacetic acid (TFA) and formic acid (FA) were purchased from Aladdin Chemistry Co., Ltd. (Shanghai, China) and Xilong Chemical Industrial Factory Co., Ltd. (Shantou, China), respectively. Sodium dodecyl sulfate (SDS) was obtained from Generay Biotech Co., Ltd (Shanghai, China). Water was purified and deionized with a Milli-Q system from Millipore (Bedford, MA, USA).

### Preparation of Stock Solutions, Calibration Standards and Quality Controls (QCs)

Protein stock solutions (0.05 mg/mL, purity >90%) were prepared in deionized water. The concentrations of FRα and FRβ were determined as 0.0493 mg/mL and 0.0478 mg/mL, respectively, using a BCA protein assay kit (Pierce Biotechnology, Inc., Rockford, IL, USA). The solutions were stored at −20 °C in a brown glass tube to protect them from light. The corresponding isotope-labeled synthetic peptides were used as internal standards. The internal standards were also weighed, and a 5 μg/mL stock solution of each internal standard was prepared in deionized water. A 60 ng/mL internal standard solution was prepared by diluting the stock solution with an ACN: water mixture (50:50, v/v) containing 0.1% FA.

FRα and FRβ calibration standards were prepared by serial diluting the stock solutions using FR-depleted cellular and tissue extracts as the matrix. The experimental details for the preparation of the immuno-depleted matrix have been provided in our previous work and are briefly given in the Supplementary Material^19,20^. A calibration curve was acquired using the standards containing both proteins. The calibration standards were prepared at 500, 1000, 2000, 3750, 5000, 7500 and 10000 ng/mL. The QC standards (i.e., lower limit of quantification (LLOQ), low QC, mid QC and high QC) were prepared at 500, 1500, 3750 and 8000 ng/mL, respectively, in the same matrix and frozen prior to use.

### Cell Culture and Tissue Collection

MCF-7/WT (ATTC, Manassas, VA) and MDA-MB-231 (ATTC, Manassas, VA) cells were cultured in DMEM supplemented with 10% fetal bovine serum, 80 U/mL penicillin and 80 μg/mL streptomycin at 37 °C and 5% CO_2_. MCF-7/ADR (Keygen Biotech, Nanjing, China) and T47D (ATTC, Manassas, VA) cells were cultured in RPMI 1640 media (with L-glutamine and sodium pyruvate) supplemented with 10% fetal bovine serum. MCF-10A cells (ATTC, Manassas, VA) were routinely maintained in DMEM/F12 supplemented with 10% fetal bovine serum and 1% penicillin/streptomycin. The cells were split every 5–7 days by the lifting cells with 0.25% trypsin, and the cells were fed between splits by the addition of fresh medium. The macrophages were isolated from fresh breast tissue with a Percoll^TM^ Density Gradient Centrifugation Media (GE Healthcare, Buckinghamshire, UK; please see the Supplementary Material). The cells were counted with a hemocytometer (Qiujing, Shanghai, China). Cell viability was assessed by Trypan blue (0.4%) exclusion. The cell suspensions, Trypan blue and 1 × PBS were mixed in a 2:5:3 ratio, and the percentage of viable cells was counted after incubation for 5 min at 37 °C.

The breast tissue collection in this study was approved by the Institutional Review Board of Nanjing Medical University. The methods were performed in accordance with the approved guidelines. Sixty pairs of breast tissue samples consisting of tumors and adjacent normal sections were collected consecutively between January 2012 and December 2012 at the First Affiliated Hospital of Nanjing Medical University and Nanjing Drum Tower Hospital, Nanjing, China (mean patient age, 52.9 ± 8.6 years; age range, 38–65 years). The tissue sections were confirmed as normal or cancerous by hospital pathologists. Among the tumor tissues, 39 samples were invasive ductal carcinoma, 19 were invasive lobular carcinoma and 2 were other histological types. The tumor grade was assessed according to the Nottingham scheme^21^. Histological evaluation of the adjacent normal tissue samples showed no indication of contamination from the tumor or other abnormal cells. The patients were biologically unrelated, but all patients belonged to the Han Chinese ethnic group from the Jiangsu province in China. Informed consent was obtained from the subjects. The tissue samples were stored at −80 °C until analysis. Prior to protein extraction, the tissue samples were thawed to room temperature and rinsed thoroughly with deionized water. The fat tissue was removed, and the remaining tissue was cut into small pieces and transferred to tubes. In addition to the LC-MS/MS analysis, the samples were also subject to ER, PR and HER2 tests. The HER2 status was evaluated according to the ASCO/CAP guidelines as a 4-grade system (0–3+). Only a 3+ HER2 score was counted as positive in breast cancer, and the ER and PR positive cut-off points were deemed positive if >1% of tumor cells were stained.

### Membrane Protein Extraction

The extraction of membrane proteins with Triton X-114 has been previously described^20^,^22^. In detail, the cells were pelleted at 1480 × g for 10 min and then resuspended in 500 μL of 1% Triton X-114 extraction buffer (1 mM DTT, 2 mM EDTA-2Na in 50 mM Tris/HCl, pH 7.4) containing 1% protease inhibitor cocktail. The samples were incubated on ice for 30 min and at 37 °C for 10 min, and then centrifuged at 10,000 × g for 3 min to separate the detergent and aqueous phases. To achieve a complete extraction, 500 μL of 1% Triton X-114 extraction buffer and 500 μl of 0.06% Triton X-114 wash buffer (1 mM DTT, 2 mM EDTA in 50 mM Tris/HCl, pH 7.4) were added to the aqueous and detergent phases, respectively. The incubation and centrifugation steps were repeated. The detergent phases were combined, and the proteins were precipitated using cold acetone. The protein pellets were then dissolved in a 1% SDS solution. The protein concentrations of the resulting membrane fractions were determined using the BCA protein assay kit. For the breast tissue samples, approximately 50 mg tissue was weighed and resuspended in buffer containing 50 mM Tris/HCl, pH 7.4, 2 mM EDTA, 1 mM DTT, 150 mM NaCl and 1% protease inhibitor cocktail. The samples were homogenized using a Bio-Gen PRO200 homogenizer (PRO Scientific Inc., Oxford, CT, USA). After centrifugation, the collected samples were treated with Triton X-114 and extracted using the procedure described above.

### In-solution Tryptic Digestion

A 100 μL aliquot of each sample (calibration standards, QCs and extracted samples) was mixed with 50 μL of 50 mM NH_4_HCO_3_ and denatured at 95 °C for 8 min. Subsequently, the protein was reduced by the addition of 50 mM DTT until a final concentration of 10 mM was achieved and incubated at 60 °C for 20 min. The sample was then alkylated by adding 400 mM IAA to obtain a final concentration of 50 mM and incubated at room temperature for 6 h in the dark. Finally, sequencing grade trypsin was added, and the sample was incubated at 37 °C for 24 h. The reaction was stopped by adding 10 μL of 0.1% TFA. Then, 100 μL of the internal standard solution was added to the tryptic peptide mixture before transferring it to an Oasis HLB cartridge (60 mg/3 mL; Waters, Milford, MA, USA) that had been preconditioned with 3 mL ACN and 3 mL deionized water. After the sample was loaded, the cartridge was washed with 2 mL of water and 2 mL of ACN: water (50:50, v/v) and eluted with 1 mL of 100% ACN. Finally, the eluent was evaporated to dryness, and the sample was resuspended in 100 μL of ACN: water (50:50, v/v) containing 0.1% FA.

### LC-MS/MS

An Agilent Series 1200 HPLC system (Agilent Technologies, Waldbronn, Germany) and a 6410 Triple Quad LC/MS mass spectrometer (Agilent Technologies, Santa Clara, CA, USA) were used for the LC-MS/MS studies.

The liquid chromatography separations were performed on a Hypersil GOLD column (3 μm, 100 mm × 2.1 mm; Thermo Fisher Scientific, USA) at room temperature. The mobile phase consisted of solvent A (0.1% FA in water) and solvent B (0.1% FA in methanol). A linear gradient with a flow rate of 0.3 mL/min was applied in the following manner: B 10% (0 min) → 10% (1 min) → 90% (4 min) → 90% (8 min) → 10% (9 min). The injection volume was 10 μL.

The mass spectrometer was interfaced with an electrospray ion source and operated in the positive MRM mode. Q1 and Q3 were both set at unit resolution. The flow of the drying gas was 10 L/min, and the drying gas temperature was held at 350 °C. The electrospray capillary voltage was optimized to 4000 V. The nebulizer pressure was set to 45 psi. The data were collected and processed using the Agilent MassHunter Workstation Software (version B.01.04).

### Conventional Analytical Methods

For the experimental details of IHC and Western blotting, please see the Supplementary Material.

### Ethical Approval

This study was approved by the Institutional Review Board of Nanjing Medical University, Nanjing, China.

## Results and Discussion

### Selection of FRα and FRβ Isoform-specific Surrogate Peptides

**Figure 1:**
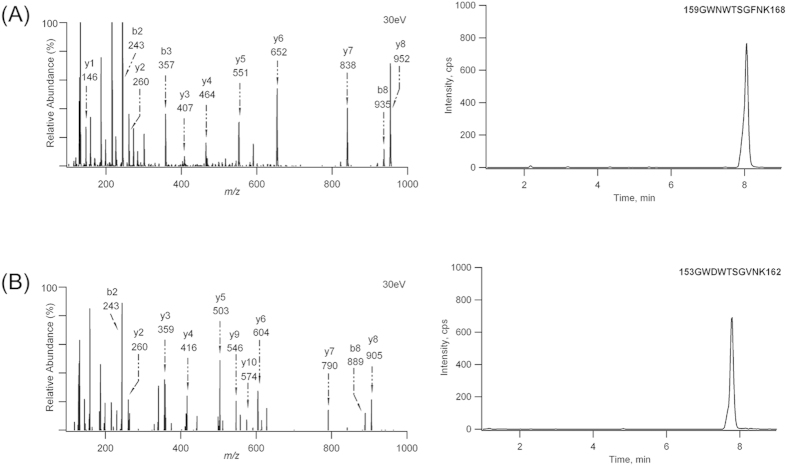
The product ion spectra and representative LC-MS/MS chromatograms for 159GWNWTSGFNK168 (**A**) and 153GWDWTSGVNK162 (**B**). The internal standard is omitted for clarity.

In complex proteomes, a major challenge frequently encountered when identifying candidate biomarkers and targets of anti-cancer therapy is to establish quantitative assays that distinguish between highly homologous proteins. Among the various assays for protein quantification, LC-MS/MS-based targeted proteomics is a particularly valuable method of choice. For a targeted proteomics assay, it is often not trivial to reconnect surrogate peptides to a precise protein of origin due to the presence of shared proteolytic peptides from protein isoforms. Thus, the most critical step in the experimental design and assay establishment for measuring protein isoforms is the selection of surrogate peptides that are isoform-specific. In this study, both FRα and FRβ are 38 kDa in size, as previously mentioned. The result of amino acid sequence alignment indicated an 80% identity between FRα and FRβ (Fig. 1S), leading to fewer available peptides that are isoform-specific and meet the general criteria for peptide selection (e.g., uniqueness and adequate response)^23^,^24^. After excluding the peptides that were either not isoform-specific or not unique to the proteins, we performed an LC-MS/MS analysis of protein digests with the list of potential peptides to determine the tryptic peptides that were present in the greatest abundance. As a result, the most abundant peptides were the doubly charged ions of 159GWNWTSGFNK168 for FRα and 153GWDWTSGVNK162 for FRβ^20^. Further evaluation using the synthetic reference peptides showed that the tryptic and reference peptides co-eluted perfectly and exhibited identical fragmentation patterns. In addition, these sequences were found to be unique to FR proteins (accession no. P15328 (FOLR1_HUMAN) and P14207 (FOLR2_HUMAN)) using a BLAST search, suggesting that they could be used to specifically quantify FRα and FRβ. The product ion spectra and extracted ion chromatograms of 159GWNWTSGFNK168 and 153GWDWTSGVNK162 are shown in Fig. 1. The characteristic sequence-specific b ions and y ions were indicative of these peptides. Therefore, synthetic stable isotope-labeled peptides, 159GWNWTSGF*NK168 and 153GWDWTSGV*NK162, were prepared. The peptides were labeled at the corresponding phenylalanine (^13^C_9_) and valine positions (D_8_) and eluted at the same retention time (7.77 min and 8.05 min) as the non-labeled peptides. The product ion spectra of the stable isotope-labeled peptides were also acquired and validated (data not shown).

### Development and Validation of an LC-MS/MS-based Targeted Proteomics Assay

The completeness and reproducibility of trypsin digestion must be carefully assessed to provide an accurate and precise amount of the proteins. Though 100% digestion efficiency is generally ideal for protein quantification, reproducible digestion is more than acceptable in this study because pure proteins were used to prepare the standards. Thus, only a brief description was given here. Following a similar process provided in our previous work^20^,^25^ and using the substrate peptide containing the same peptide sequence (to mimic a piece of the targeted protein), the digestion efficiency was calculated by comparing the response ratios of the tryptic peptide after digestion and the equimolar synthetic peptide standard in the digestion. The estimated values were 97.2% and 95.4% for 159GWNWTSGFNK168 and 153GWDWTSGVNK162, respectively.

**Figure 2:**
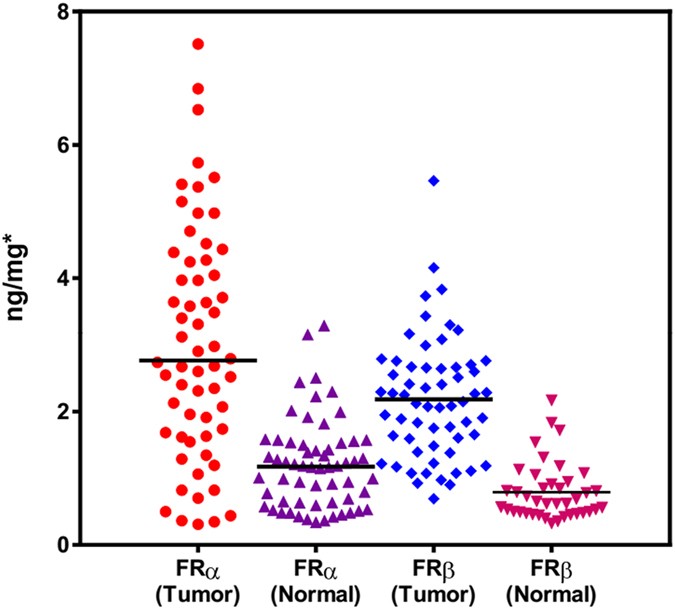
The levels of FR isoforms in 60 matched pairs of breast tissue samples.

**Figure 3:**
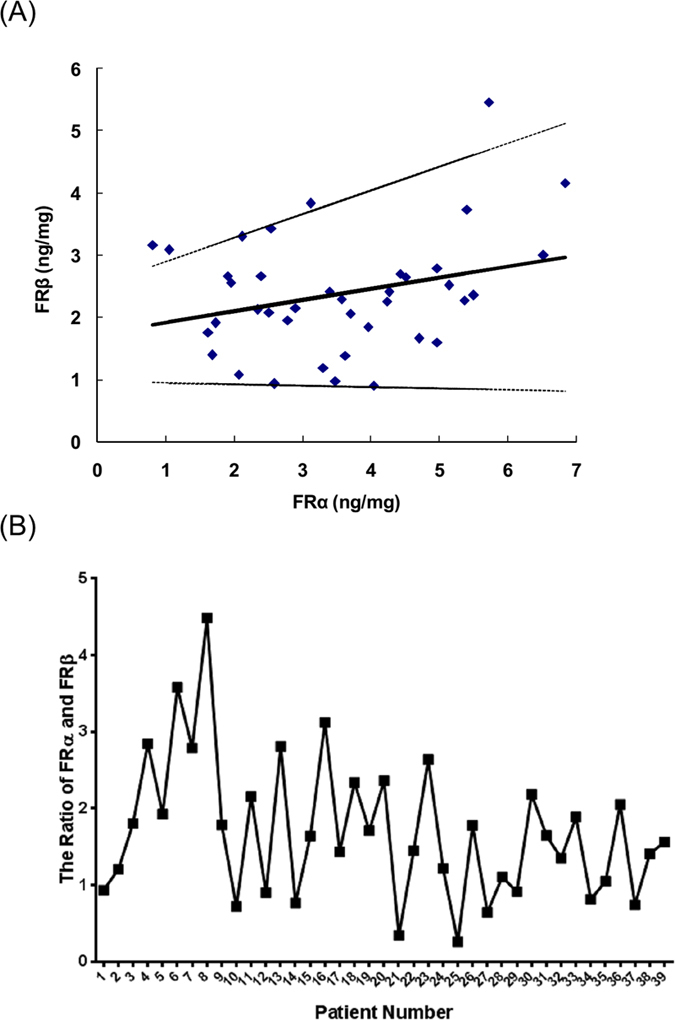
The ratio of FRα to FRβ in breast cancer patients (**A**) and the regression analysis of FRα with FRβ (**B**). The solid line corresponds to the regression line. Dashed lines represent the 95% confidence interval for the regression line.

**Figure 4:**
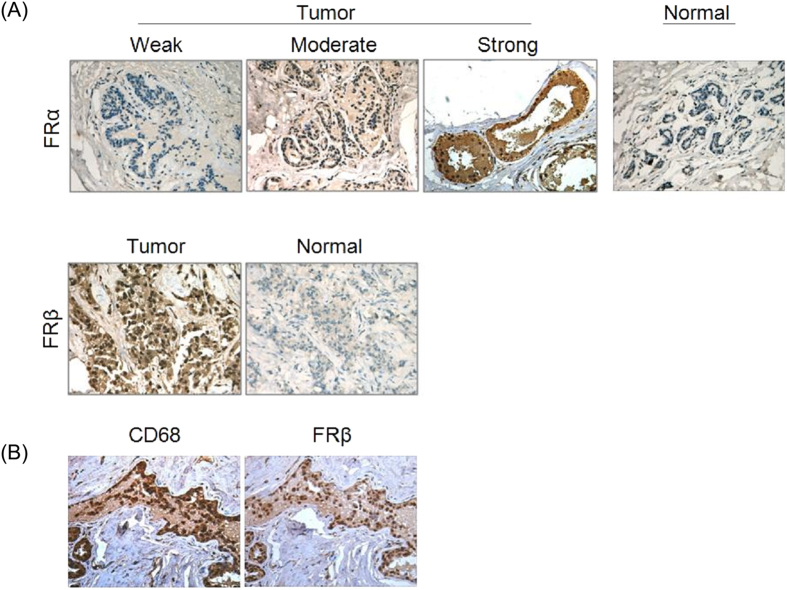
IHC images of representative breast tumors with weak, moderate and strong FRα expression and normal tissue (**A**); FRβ expression in tumor tissues where CD68^+^ is present (**B**).

**Figure 5:**
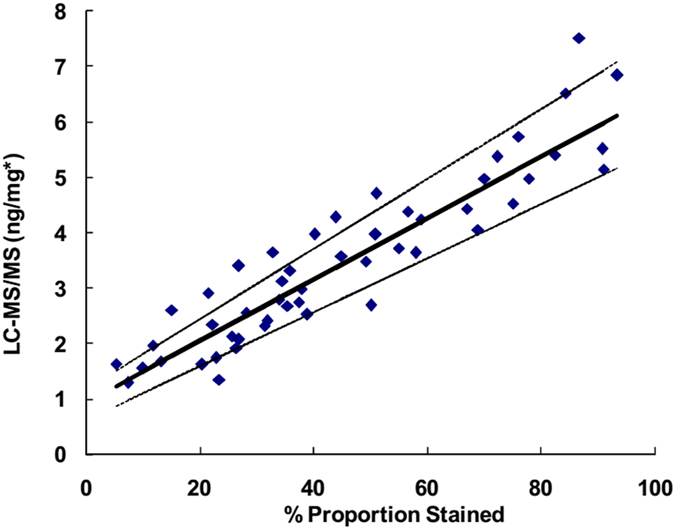
Passing-Bablok regression analysis for the IHC *vs.* the LC-MS/MS assay. The solid line corresponds to the regression line. Dashed lines represent the 95% confidence interval for the regression line. *FRα ng/mg breast tissue.

Another important step in protein quantification is to generate high-quality MRM^26^. In this study, the transitions that gave the best signal-to-noise and limit of quantification (LOQ) for 159GWNWTSGFNK168 and 153GWDWTSGVNK162 were both afforded by the product ion b2 *m/z* 243.9. These characteristic mass patterns were also observed using the stable isotope-labeled internal standards. Using the transitions of *m/z* 598.7 → 243.9, *m/z* 603.4 → 243.9 (IS) and *m/z* 575.2 → 243.9, *m/z* 579.3 → 243.9 (IS), an LC-MS/MS assay was developed and validated to measure FRα and FRβ. Solid phase extraction was selected as the technique of choice for sample cleanup and enrichment in this study because, as previously reported, it has shown great promise for sample preparation^25^. Calibration standards containing both proteins were prepared to reduce the analysis time^7^. The calibration curves were constructed using a weighted linear regression model with a weighting factor of 1/x^2^. The relative peak area ratio of the analyte and the stable isotope-labeled internal standard was plotted against concentration. Because similar results were obtained for the cellular and tissue extracts, only the validation for the tissue samples was presented. Representative calibration curves are shown in Fig. 2S. The LOQ was 500 ng/mL. Chromatograms of the LLOQs and blanks are shown in Fig. 3S. Notably, the LLOQ in the present study was the lowest standard on the calibration curve if the following conditions were met: 1) the analyte response at the LLOQ should be at least 5 times the response compared to blank response and 2) analyte response should be identifiable, discrete, and reproducible with a precision of 20% and accuracy of 80–120%^27^,^28^. The results indicated that no significant interfering peak was found at the retention time for peptides in the chromatogram of the blank matrix, in agreement with the condition that the analyte response at the LLOQ should be at least 5 times the blank response. This outcome demonstrated that the extent of depletion was significant in the immuno-depleted membrane fraction, which was also confirmed by Western blotting (Fig. 4S). Thus, the FR-depleted membrane fraction can be utilized as a surrogate matrix in this study. To further examine the specificity of the MRM transitions for quantification, a second product ion of the peptides was evaluated here for confirmation (i.e., *m/z* 598.7 → 261.1 and *m/z* 575.2 → 358.9)^29^,^30^. The values determined for the MRM transition pairs of each peptide were consistent for each standard and throughout the calibration range (Table 1S). After confirmation, the transitions of *m/z* 598.7 → 243.9 (FRα) and *m/z* 575.2 → 243.9 (FRβ) were employed for sample analysis. It is worth mentioning that utilization of MRM transitions with more than one fragment ion for one precursor is often as a confirmative measure^31^,^32^, but it can increase the detection specificity of peptides derived from low-abundant proteins in some cases^33^,^34^. Because the quantification values for a single protein could be surrogate peptide-dependent^30^,^35^, a second surrogate peptide under a single condition was used for additional confirmation^29^. Due to the absence of an appropriate second peptide for FRβ, only FRα was processed using another surrogate peptide 77DVSYLYR83 (Fig. 5S), and the result will be evaluated in the next section.

The precision and accuracy of the assay were assessed by observing the response of the QC samples with four different concentrations of peptides in three validation runs. The intra- and inter-day precisions were expressed as the percent coefficient of variation (%CV). The accuracy was obtained by comparing the averaged calculated concentrations to their nominal values (%bias). The results are listed in Table 2S. Both accuracy and precision were ≤ ± 15% (LLOQ, ≤ ± 20%)^28^.

Three freeze-thaw cycles, 48 h post-preparative (4 °C) and 12 h room temperature stabilities were also conducted here. The results indicated that the stability of the peptides and proteins was acceptable (data not shown).

### Simultaneous Quantification of FRα and FRβ in Breast Tissue Samples

The LC-MS/MS-based targeted proteomics assay developed and validated above was first applied to the detection and quantification of FR isoforms in human breast tissue samples. Sixty matched pairs of breast tissue samples were subjected to analysis. In the normal tissue, the levels of FRα and FRβ were accurately quantified as 1.20 ± 0.66 ng/mg breast tissue (range: 0.34–3.29 ng/mg) and 0.79 ± 0.43 ng/mg (range: 0.33–2.17 ng/mg), respectively, and in the tumor tissue, the levels of FRα and FRβ were 3.02 ± 1.73 ng/mg (range: 0.31–7.51 ng/mg) and 2.18 ± 0.90 ng/mg (range: 0.69–5.46 ng/mg), respectively (Fig. 2). The FRα result was not significantly different from that using 77DVSYLYR83 as surrogate peptide. Notably, FRβ was undetectable in the normal tissue from 19 patients (32%, <LOQ). A two-way comparison using the Mann-Whitney test showed that normal tissue samples have significantly lower levels of the FR isoforms compared to the tumors (P < 0.0001). The FRα and FRβ reference intervals were calculated as −0.104 (95% CI, −0.345–0.137) ng/mg to 2.49 (95% CI, 2.25–2.73) ng/mg, and −0.046 (95% CI, −0.240–0.148) ng/mg to 1.63 (95% CI, 1.43–1.82) ng/mg, respectively, using MedCalc® software Version 11.6.1 and data from the normal tissue. Thus, 35 and 46 of the 60 tumor samples contained FRα and FRβ levels, respectively, and exceeded their estimated reference intervals. Compared to FRα, FRβ expression was more pronounced and less variable in breast tumors, implying that FRβ may be a better candidate for certain patients.

Furthermore, an evaluation of the patients’ breast cancer histology indicated that both invasive ductal carcinoma (IDC) and invasive lobular carcinoma (ILC) had an enhanced level of the FRs, and the increase of these receptor proteins was more significant in IDC (Table 1). Because the frequency and degree of FR expression were likely associated with the cancer characteristics, their levels were also compared according to the clinical histopathological features (*i.e.*, tumor size, tumor grade, and lymph node status) and molecular subtypes of IDC (Table 2). The FRα result indicated that the histopathological characteristics may be correlated to its expression, which was in agreement with previous reports^36^. However, the obtained differences were not statistically significant (p > 0.05). More importantly, we found that FRα was negatively associated with PR/ER and HER2 expression in breast cancer. A possible explanation could be that the hormone receptor-positive tumors often had a lower incidence of FRα. There is evidence indicating that FRα expression is regulated by steroid hormones, particularly estrogens^37^. Specifically, 7β-estradiol can down-regulate FRα expression by direct action of ER on the P4 promoter of FRα. Thus, it is not surprising to also find enriched FRα in clinical triple negative breast cancer (TNBC) patients^36^. The previous result that IDC patients tended to have enhanced FRα may be explained because triple negative breast cancer (TNBC) is usually invasive and usually begins in the breast ducts^38^.

Different from FRα, metastasis was the only factor closely associated with the level of FRβ in this study. Because the occurrence of FRβ in breast tumors remained undetected until recently, the role of FRβ in disease development has been rarely reported. Based on the previous findings, FRβ is expressed by TAMs that enhanced tumor progression by promoting tumor invasion, migration and angiogenesis^25^,^39^. Thus, TAMs were often abundantly present in malignant tumors, as should be FRβ. While macrophage infiltration density is also reported to be strongly associated with ER/PR and HER2 status^39^,^40^, we found that these hormones were not significantly associated with FRβ.

The data show that the FRα and FRβ levels in individual patients do not fluctuate in concert (Fig. 3A). Further regression analysis revealed that there was a positive relationship between FRα and FRβ, whereas the obtained correlation coefficient (r = 0.285, p > 0.05) suggested that this relationship was low (Fig. 3B). Despite their differential tissue distribution, the expression ratio of FRβ to FRα was still processed across the patient samples in this study. As a result, most samples indicated a substantial difference in the pairwise ratio of the FR isoforms. Therefore, the ratio of the FR isoforms may have the potential to stratify breast cancer patients into subgroups, which deserves further testing with a larger sample size.

### Comparison of Targeted Proteomics and IHC for FR Isoform Analysis

In general, the proteins in tissue samples are typically determined using IHC. From the IHC images (Fig. 4), we should note that one of its advantages is the capability to discern between tumors containing a small subpopulation of FR-positive cells and those showing a low level of FR in 100% of the cells^41^. Moreover, the co-localization of FRβ and CD68, which is frequently used as a marker of macrophages, proved once again that FRβ occurred in TAMs from the stroma, but not in epithelial cells.

However, the IHC assays are normally not quantitative, are difficult to standardize across laboratories and are subject to an inherent intra- and inter-observer variability, despite tremendous efforts to establish guidelines and protocols^42^. The subjective counting of stained cells in IHC (e.g., 0, no membrane staining; 1+, faint partial; 2+, weak-to-moderate; 3+, intense complete) could cause large deviations in cancer diagnosis, treatment and monitoring. In addition, FRs are also expressed in normal breast tissue to a certain degree^43^. Most importantly, current immunohistochemical methods to study the expression of multiple proteins or protein isoforms in a single tissue section suffer from several limitations (e.g., cross-reactivity of antibodies), even though several antigen retrieval/unmasking methods have been developed^44^. Alternatively, single immunohistochemical stains in which multiple sections are cut from a tissue sample can be performed, and each section is stained for a different protein. However, each tissue section contains different cells, which may be problematic in some situations.

In this study, we compared the FRα result of the LC-MS/MS-based targeted proteomics to that obtained from IHC. Although the immunostaining was not scored, the percent positive cells (%) were counted for evaluation in this study^45^. Ten samples showed no staining, whereas targeted proteomics provided the quantitative values for all of the samples. The methods were compared using a Passing-Bablok regression analysis, which was performed using the statistical program MedCalc version 12.7.4 (MedCalc Software). As shown in Fig. 5, the LC-MS/MS method was comparable to IHC (y = 0.055 x + 0.931, with no significant deviation from linearity (P = 0.68)). The estimated confidence intervals of the slope and intercept were (0.049–0.063) and (0.608–1.170), respectively. Notably, the values determined in targeted proteomics are no longer subjective and can easily be used for reference range establishment and further substratification according to the clinical histopathological features.

### Quantification of FRα and FRβ in Breast Cells and TAMs Isolated from Breast Tumors

Real clinical samples, such as breast tissue, are so complex that the tumor cells and other cell types where FRα and FRβ may be present are not easily separated. These different cell types represent variable portions of the whole tissue and may also exhibit heterogeneity in terms of the level and type of FR expressed. To determine the possible cell type-specific FR isoform expression, we quantified the FR isoforms in a number of human breast cells (i.e., the MCF-10A normal cell line, the MCF-7/WT, T47D and MDA-MB-231 drug-sensitive cancer cell lines, and the MCF-7/ADR drug-resistant cancer cell line) using the LC-MS/MS-based targeted proteomics assay. The results are shown in Table 3. Notably, several studies have investigated the expression of FRα in breast cells; however, the results of these studies are conflicting. Some of them reported its presence in one or more cell types^20^,^46^, while others did not^47^. In this report, we detected FRα at variable levels in all tested cell lines, and FRα was overexpressed in the cancer cells compared to the normal cells. In addition, the MDA-MB-231 cells had the greatest abundance of this FR isoform. With regard to the characteristics of the cell lines, the T47D and MCF-7 cells are ER+/PR+/HER2-, and the MDA-MB-231 cells are triple negative (ER-/PR-/HER2-, Table 3S). Thus, it is theoretically proposed that FRα expression is closely related to the ER/PR status of the tumor cells. This phenomenon was in accord with the positive association between FRα and ER- observed in tissue, perhaps more importantly with TNBC^36^. Because the MDA-MB-231 cell line is also a highly invasive and metastatic cell line, the metastatic activity of cells may be another factor that could be associated with the FRα level. Moreover, the enhanced FRα expression in the MCF-7/ADR cells implied that this protein receptor may also be involved in the response of tumor cells to anticancer drugs and the acquisition of drug resistance.

In contrast, FRβ was not detected in any of the tested cell lines. The failure to detect this FR isoform suggests that it is either not present or is present at much lower levels in these breast cells. Taking into account the observation of FRβ in breast tissue, FRβ should exist in cells other than cancer cells, such as macrophages. Thus, fresh breast tumor and adjacent normal tissue samples were pooled and fractionated. The macrophage-enriched cell populations expressed FRβ. In addition, FRβ was more abundant in TAMs (57.2 ± 4.6 fg/cell) than macrophages from normal tissue (15.6 ± 1.4 fg/cell). In fact, the macrophages in the normal tissue were primarily in a resting state and did not express FRβ. The resting macrophages can become activated by stimulation such as disease. Active macrophages such as TAMs could have an elevated level of FRβ on their surface, and accumulated at the sites of inflammation and in tumors. In this study, we first provided quantitative evidence of the existence of FRβ in macrophages. Subsequently, the level of FRβ was determined for an individual breast tumor. The varying amounts of FRβ in 5 TAM samples may imply its potential role as a biomarker for defining breast cancer subtypes (Table 4S).

To further evaluate the LC-MS/MS performance, the amount of FRs in cells was also examined using Western blotting (Fig. 6S). Compared with the qualitative/semi-quantitative results and the limited sensitivity of antibody-based method, the targeted proteomics approach provided the quantitative amounts of proteins.

## Conclusions

In this report, the developed LC-MS/MS-based targeted proteomics assay allowed the simultaneous and accurate quantification of FR isoforms (i.e., FRα and FRβ) in biological samples. Using multiplexed isoform-specific MRM transitions, these isoforms can be quantified by ignoring their homology and real locations. In addition to demonstrating FRα overexpression in breast cancer cells and tissue samples, the abundance of FRβ in TAMs from the tumor-associated stroma was also indicated using this assay. Moreover, the findings provided evidence for the association between the level of FR isoforms and several histopathological features and molecular subtypes. Thus, this isoform-based quantitative information may lead to better cancer patient stratification with diagnostic significance. However, additional, larger patient cohorts must be tested to determine whether FRα and FRβ can be used alone as independent factors or in combination with other markers, and whether they are more selective for specific clinical applications in breast cancer diagnosis and treatment. Nevertheless, targeted proteomics shows potential for future studies to clarify the isoform-specific clinical significance of the FR family and also other protein isoform families.

## Additional Information

**How to cite this article**: Yang, T. *et al.* Targeted Proteomics Enables Simultaneous Quantification of Folate Receptor Isoforms and Potential Isoform-based Diagnosis in Breast Cancer. *Sci. Rep.* **5**, 16733; doi: 10.1038/srep16733 (2015).

## Supplementary Material

Supplementary Material

## Figures and Tables

**Table 1:** The level of FR isoforms in breast cancer patients.

Histology	Cases	FRα (ng/mg)			FRβ (ng/mg)		
		Tumor	Normal	*p*^a^	Tumor	Normal	*p*^a^
Invasive Ductal Carcinoma	39	3.54 ± 1.51	1.18 ± 0.57	<0.0001	2.25 ± 1.04	0.80 ± 0.44	< 0.0001
Invasive Lobular Carcinoma	19	2.22 ± 1.79	1.32 ± 0.85	0.0234	2.06 ± 0.93	0.75 ± 0.40	0.0037
Others	2	1.45 ± 0.14	1.30 ± 0.03	0.434	—	—	—

*P* value of less than 0.05 was considered statistically significant.

^a^by paired t-test.

**Table 2:** Correlation of the level of FR isoforms with the histopathological parameters and molecular subtypes of invasive ductal carcinoma.

Parameters		Cases	FRα (ng/mg) in Tumor	p^a^	FRβ (ng/mg) in Tumor	p^a^
Tumor Size	≤2 cm	6	3.05 ± 1.43	0.491	1.89 ± 0.98	
	2–5 cm	27	3.56 ± 1.60		2.46 ± 0.99	
	> 5 cm	6	3.92 ± 1.24		2.49 ± 0.70	0.46
Lymph Node Status	N0	4	2.93 ± 1.53	0.217	1.72 ± 0.24	
	N1	10	3.07 ± 1.05		1.76 ± 0.83	
	N2	15	3.44 ± 1.62		2.45 ± 0.66	
	N3	10	3.62 ± 1.73		3.15 ± 1.06	0.009
Tumor Grade	GI	10	3.15 ± 1.25	0.152	1.89 ± 0.67	
	GII	15	3.29 ± 1.50		2.33 ± 0.85	
	GIII	14	4.41 ± 1.56		2.94 ± 1.06	0.078
Molecular Subtype	PR/ER+	22	2.92 ± 1.21	0.003	2.16 ± 0.85	
	PR/ER−	17	3.93 ± 1.52		2.64 ± 1.04	0.32
	7	HER2+	2.52 ± 0.94	0.031	1.91 ± 0.78	
	HER2−	32	3.76 ± 1.52		2.48 ± 0.97	0.188

*P* value of less than 0.05 was considered statistically significant.

^a^By Kruskal-Wallis test or Mann-Whitney test.

**Table 3:** FR isoform expression in breast cells and macrophages isolated from fresh breast tissue.

FR Isoforms		MCF-10A	MCF-7/WT	T47D	MDA-MB-231	MCF-7/ADR	TAMs	Macrophages in Normal Tissue
FRα	(fg/cell)	12.8 ± 0.9	209 ± 5	138 ± 7	369 ± 6	286 ± 11	—	—
	(amol/cell)	0.341 ± 0.023	5.51 ± 0.14	3.62 ± 0.19	9.72 ± 0.17	7.54 ± 0.29	—	—
FRβ	(fg/cell)	—	—	—	—	—	57.2 ± 4.6	15.6 ± 1.4
	(amol/cell)	—	—	—	—	—	1.51 ± 0.12	0.591 ± 0.053

## References

[b1] Feng Y. *et al.* A folate receptor beta-specific human monoclonal antibody recognizes activated macrophage of rheumatoid patients and mediates antibody-dependent cell-mediated cytotoxicity. Arthritis Res Ther 13, R59 (2011). 21477314 10.1186/ar3312 PMC3132054

[b2] Elnakat H. & Ratnam M. Distribution, functionality and gene regulation of folate receptor isoforms: implications in targeted therapy. Adv Drug Deliv Rev 56, 1067–84 (2004). 15094207 10.1016/j.addr.2004.01.001

[b3] Ross J. F. *et al.* Folate receptor type β is a neutrophilic lineage marker and is differentially expressed in myeloid leukemia. Cancer 85, 348–357 (1999). 10023702 10.1002/(sici)1097-0142(19990115)85:2<348::aid-cncr12>3.0.co;2-4

[b4] Wu M., Gunning W. & Ratnam M. Expression of folate receptor type alpha in relation to cell type, malignancy, and differentiation in ovary, uterus, and cervix. Cancer Epidemiol Biomarkers Prev 8, 775–82 (1999). 10498396

[b5] Puig-Kroger A. *et al.* Folate receptor beta is expressed by tumor-associated macrophages and constitutes a marker for M2 anti-inflammatory/regulatory macrophages. Cancer Res 69, 9395–403 (2009). 19951991 10.1158/0008-5472.CAN-09-2050

[b6] Arendt L. M., Rudnick J. A., Keller P. J. & Kuperwasser C. Stroma in breast development and disease. Semin Cell Dev Biol 21, 11–8 (2010). 19857593 10.1016/j.semcdb.2009.10.003 PMC2823823

[b7] Ronnov-Jessen L., Petersen O. W. & Bissell M. J. Cellular changes involved in conversion of normal to malignant breast: importance of the stromal reaction. Physiol Rev 76, 69–125 (1996). 8592733 10.1152/physrev.1996.76.1.69

[b8] Tucker T. G., Milne A. M., Fournel-Gigleux S., Fenner K. S. & Coughtrie M. W. Absolute immunoquantification of the expression of ABC transporters P-glycoprotein, breast cancer resistance protein and multidrug resistance-associated protein 2 in human liver and duodenum. Biochem Pharmacol 83, 279–85 (2012). 22062654 10.1016/j.bcp.2011.10.017

[b9] Tsukamoto F. *et al.* Immunohistochemical Detection of P-glycoprotein in Breast Cancer and Its Significance as a Prognostic Factor. Breast Cancer 4, 259–263 (1997). 11091611 10.1007/BF02966518

[b10] Deb S. *et al.* In Methods in Molecular Biology, Vol. 962 (eds Deb S. *et al.* ) 15–29 (Humana Press, 2013).

[b11] Barnidge D. R. *et al.* Absolute quantification of the G protein-coupled receptor rhodopsin by LC/MS/MS using proteolysis product peptides and synthetic peptide standards. Anal Chem 75, 445–51 (2003). 12585469 10.1021/ac026154+

[b12] Jahan-Tigh R. R., Ryan C., Obermoser G. & Schwarzenberger K. Flow cytometry. J Invest Dermatol 132, e1, 1–6 (2012). 10.1038/jid.2012.282 22971922

[b13] Cox J. & Mann M. Quantitative, high-resolution proteomics for data-driven systems biology. Annu Rev Biochem 80, 273–99 (2012). 21548781 10.1146/annurev-biochem-061308-093216

[b14] Doerr A. Targeted proteomics. Nature Methods 8, 43 (2011).

[b15] Kim Y. J., Gallien S., van Oostrum J. & Domon B. Targeted proteomics strategy applied to biomarker evaluation. Proteomics Clin Appl 7, 739–47 (2013). 24123942 10.1002/prca.201300070

[b16] Tang H. Y. *et al.* Protein isoform-specific validation defines multiple chloride intracellular channel and tropomyosin isoforms as serological biomarkers of ovarian cancer. J Proteomics 89, 165–78 (2013). 23792823 10.1016/j.jprot.2013.06.016 PMC3779132

[b17] Peng Y. *et al.* Top-down targeted proteomics for deep sequencing of tropomyosin isoforms. J Proteome Res 12, 187–98 (2013). 23256820 10.1021/pr301054n PMC3596867

[b18] Flood-Nichols S. K. *et al.* Longitudinal analysis of maternal plasma apolipoproteins in pregnancy: a targeted proteomics approach. Mol Cell Proteomics 12, 55–64 (2013). 23059768 10.1074/mcp.M112.018192 PMC3536909

[b19] Yuan Z.-Y., Luo R.-Z., Peng R.-J., Wang S.-S. & Xue C. High infiltration of tumor-associated macrophages in triple-negative breast cancer is associated with a higher risk of distant metastasis. OncoTargets and therapy 7, 1475–1480 (2014). 25187727 10.2147/OTT.S61838 PMC4149399

[b20] Pan W. *et al.* Dual-targeted nanocarrier based on cell surface receptor and intracellular mRNA: an effective strategy for cancer cell imaging and therapy. Anal Chem 85, 6930–5 (2013). 23772649 10.1021/ac401405n

[b21] Elston C. W. & Ellis I. O. Pathological prognostic factors in breast cancer. I. The value of histological grade in breast cancer: experience from a large study with long-term follow-up. Histopathology 19, 403–10 (1991). 1757079 10.1111/j.1365-2559.1991.tb00229.x

[b22] Pryde J. G. Partitioning of Proteins in Triton X-114 (Humana Press Inc., New Jersey, 1998). 10.1385/0-89603-487-9:23 9664295

[b23] Kamiie J. *et al.* Quantitative atlas of membrane transporter proteins: development and application of a highly sensitive simultaneous LC/MS/MS method combined with novel in-silico peptide selection criteria. Pharm Res 25, 1469–83 (2008). 18219561 10.1007/s11095-008-9532-4

[b24] Uchida Y. *et al.* A study protocol for quantitative targeted absolute proteomics (QTAP) by LC-MS/MS: application for inter-strain differences in protein expression levels of transporters, receptors, claudin-5, and marker proteins at the blood-brain barrier in ddY, FVB, and C57BL/6J mice. Fluids Barriers CNS 10, 21 (2013). 23758935 10.1186/2045-8118-10-21 PMC3691662

[b25] Yuan Z.-Y., Luo R.-Z., Peng R.-J., Wang S.-S. & Xue C. High infiltration of tumor-associated macrophages in triple-negative breast cancer is associated with a higher risk of distant metastasis. OncoTargets and therapy 7, 1475–1480 (2014). 25187727 10.2147/OTT.S61838 PMC4149399

[b26] Picotti P. & Aebersold R. Selected reaction monitoring-based proteomics: workflows, potential, pitfalls and future directions. Nat Methods 9, 555–66 (2012). 22669653 10.1038/nmeth.2015

[b27] Absciex, Defining Lower Limits of Quantitaion (2010), Available at: http://www.absciex.com/Documents/Downloads/Literature/mass-spectrometry-cms_059150.pdf. (Date of access: 10th October 2015).

[b28] Biopharmaceutics Coordinating Committee in CDER, Guidance for industry-bioanalytical method validation, (2001) Available at: http://www.fda.gov/downloads/Drugs/GuidanceComplianceRegulatoryInformation/Guidances/ucm070107.pdf. (Date of access: 28th May 2015).

[b29] Balogh L. M., Kimoto E., Chupka J., Zhang H. & Lai Y. membrane protein quantification by peptide-based mass spectrometry approaches: studies on the organic anion-transporting polypeptide family. Proteomics & Bioinformatics S4, 1–8 (2012).

[b30] Kuhn E. *et al.* Quantification of C-reactive protein in the serum of patients with rheumatoid arthritis using multiple reaction monitoring mass spectrometry and 13C-labeled peptide standards. Proteomics 4, 1175–86 (2004). 15048997 10.1002/pmic.200300670

[b31] Hagman C. *et al.* Absolute quantification of monoclonal antibodies in biofluids by liquid chromatography-tandem mass spectrometry. Anal Chem 80, 1290–6 (2008). 18217771 10.1021/ac702115b

[b32] Lesur A., Varesio E. & Hopfgartner G. Accelerated tryptic digestion for the analysis of biopharmaceutical monoclonal antibodies in plasma by liquid chromatography with tandem mass spectrometric detection. J Chromatogr A 1217, 57–64 (2010). 19939394 10.1016/j.chroma.2009.11.011

[b33] van den Broek I., Niessen W. M. A. & van Dongen W. D. Bioanalytical LC-MS/MS of protein-based biopharmaceuticals. Journal of Chromatography B 929, 161–179 (2013). 10.1016/j.jchromb.2013.04.030 23685427

[b34] Duan X., Abuqayyas L., Dai L., Balthasar J. P. & Qu J. High-throughput method development for sensitive, accurate, and reproducible quantification of therapeutic monoclonal antibodies in tissues using orthogonal array optimization and nano liquid chromatography/selected reaction monitoring mass spectrometry. Anal Chem 84, 4373–82 (2012). 22519810 10.1021/ac2034166 PMC3352998

[b35] Brun V. *et al.* Isotope-labeled protein standards: toward absolute quantitative proteomics. Mol Cell Proteomics 6, 2139–49 (2007). 17848587 10.1074/mcp.M700163-MCP200

[b36] O'Shannessy D. J., Somers E. B., Maltzman J., Smale R. & Fu Y. S. Folate receptor alpha (FRA) expression in breast cancer: identification of a new molecular subtype and association with triple negative disease. Springerplus 1, 22 (2012). 23961352 10.1186/2193-1801-1-22 PMC3725886

[b37] Kelley K. M., Rowan B. G. & Ratnam M. Modulation of the folate receptor alpha gene by the estrogen receptor: mechanism and implications in tumor targeting. Cancer Res 63, 2820–8 (2003). 12782587

[b38] Kandil D. & Khan A. Triple negative breast carcinoma: the good, the bad and the ugly. Diagnostic Histopathology 18, 210–216 (2012).

[b39] Medrek C., Ponten F., Jirstrom K. & Leandersson K. The presence of tumor associated macrophages in tumor stroma as a prognostic marker for breast cancer patients. BMC Cancer 12, 306 (2012). 22824040 10.1186/1471-2407-12-306 PMC3414782

[b40] Tang X. Tumor-associated macrophages as potential diagnostic and prognostic biomarkers in breast cancer. Cancer Letters 332, 3–10 (2013). 23348699 10.1016/j.canlet.2013.01.024

[b41] Schoenherr R. M. *et al.* Multiplexed quantification of estrogen receptor and HER2/Neu in tissue and cell lysates by peptide immunoaffinity enrichment mass spectrometry. Proteomics 12, 1253–60 (2012). 22577026 10.1002/pmic.201100587 PMC3418804

[b42] Hochhauser D. & Harris A. L. Drug resistance. Br Med Bull 47, 178–96 (1991). 1650621 10.1093/oxfordjournals.bmb.a072454

[b43] Vermeulen J. F. *et al.* Immunophenotyping invasive breast cancer: paving the road for molecular imaging. BMC Cancer 12, 240 (2012). 22695343 10.1186/1471-2407-12-240 PMC3430576

[b44] van den Brand M. *et al.* Sequential immunohistochemistry: a promising new tool for the pathology laboratory. Histopathology 65, 651–7 (2014). 24766252 10.1111/his.12446

[b45] Shaikh A. *et al.* Comparison between immunoturbidimetry, size-exclusion chromatography, and LC-MS to quantify urinary albumin. Clin Chem 54, 1504–10 (2008). 18617580 10.1373/clinchem.2008.107508 PMC3903150

[b46] Chen H., Ahn R., Van den Bossche J., Thompson D. H. & O’Halloran T. V. Folate-mediated intracellular drug delivery increases the anticancer efficacy of nanoparticulate formulation of arsenic trioxide. Mol Cancer Ther 8, 1955–63 (2009). 19567824 10.1158/1535-7163.MCT-09-0045 PMC3098497

[b47] Jhaveri M. S., Rait A. S., Chung K. N., Trepel J. B. & Chang E. H. Antisense oligonucleotides targeted to the human alpha folate receptor inhibit breast cancer cell growth and sensitize the cells to doxorubicin treatment. Mol Cancer Ther 3, 1505–12 (2004). 15634643

