# Supplementary material for: Targeted Proteomics Enables Simultaneous Quantification of Folate Receptor Isoforms and Potential Isoform-based Diagnosis in Breast Cancer
Source: Sci Rep. 2015 Nov 17;5:16733. doi: 10.1038/srep16733 (PMC4648081; doi:10.1038/srep16733)
Supplement: Supplementary Material [file srep16733-s1.doc]

**Title:** Targeted Proteomics Enables Simultaneous Quantification of Folate Receptor Isoforms and Potential Isoform-based Diagnosis in Breast Cancer

**Authors:** Ting Yang, Feifei Xu, Danjun Fang, Yun Chen*

**Supplementary Tables**

Table 1S. The ratio of response for product ions of each peptide in FR-depleted membrane fraction of breast tissue.

Table 2S. Accuracy and precision for the QC samples.

Table 3S. Characteristics of breast cancer cell lines.

Table 4S. The amounts of FRβ in 5 individual TAM samples and the corresponding macrophages of adjacent normal tissue.

**Supplementary Figures**

**(A)**

**(B)**

**Figure 1S.** Aligned amino acid sequences of FRα and FRβ using Blast (A). The tryptic sites of FRs are then highlighted in blue and in bold, and the potential isoform-specific peptide candidates are underlined (B).

**Figure 2S.** Calibration curves of 159GWNWTSGFNK168 (A) and 153GWDWTSGVNK162 (B).

**Figure 3S.** The representative LC-MS/MS chromatograms of LLOQ and blank of 159GWNWTSGFNK168 and 153GWDWTSGVNK162

**Figure 4S.** The Western blotting image for FR depleted tissue extract.

**Figure 5S.** The representative LC-MS/MS chromatogram of LLOQ of 77DVSYLYR83 for FRα. The MRM transition of *m/z* 458.2→214.9 was used.

**Figure 6S.** Western images of FRα and FRβ in human breast cell lines and macrophages isolated from breast cancer and adjacent normal tissue samples. The analysis is normalized with actin.

**Supplementary Material**

S1. Immuno-depletion of Membrane Fraction

Membrane fractions were added at protein concentrations of 2 mg/ml to BioMagPlus Goat anti-Mouse IgG beads (Bangs Laboratories, Fisher, Indiana, USA) that had been pre-incubated with anti-FR antibodies (i.e., anti-FRα antibody (Abcam, Cambridge, UK) and anti-FRβ antibody (Biorbyt, Cambridge, UK)). Samples were incubated at 4°C for 60 min with gentle rotation, and then placed in a magnetic field (Magnetic separator; Bangs Laboratories, Fishers, Indiana, USA) for 2 min. Supernatants were collected and subjected to the depletion protocol a second time. The resulting supernatants were then analyzed by Western blotting using the anti-FR antibodies (Fig. 4S).

S2. Macrophage Isolation

Fresh breast tissue was weighed and transferred to a small dish that contained DMEM media. After cutting the tissue into small pieces, the sample was added with 0.25% trypsin and incubated at 37°C for 3 min. Then, DMEM media supplemented with 10% fetal bovine serum was added to stop digestion. The tissue suspension was pelleted at 1500 rpm for 5 min and resuspended in a small volume of DMEM media supplemented with 10% fetal bovine serum. After sieving, TAMs were isolated using a percoll gradient solution.

S3. Conventional Analytical Methods

S3.1 Western Blotting

Membrane extract was separated by 12% SDS-PAGE and transferred onto a polyvinylidene difluoride (PVDF) membrane (Millipore, Billerica, MA, USA). The membrane was then blocked in 5% milk overnight and incubated with an anti-FR antibody (1:250) followed by HRP-conjugated goat anti-mouse IgG (1:5000). FRs were detected using enhanced chemiluminescence reagent (Pierce, Rockford, IL, USA) according to the manufacturer’s protocol.

S3.2 IHC

Frozen sections (4 μm) were cut on a freezing microtome and pre-incubated in 5% BSA for 20 min to block nonspecific binding. Tissue sections were then incubated with anti-FR antibody (1:100) for 1 h at room temperature. The sections were washed with PBS and incubated with Powervision poly-HRP anti-mouse (1:300) for 30 min at room temperature. Five images were acquired from randomly selected areas in each slide. The staining results were compared to normal breast tissue; stains were deemed positive when clear membranous staining was observed and when expression in tumor tissue samples was clearly higher than in normal ones. The proportion of FR-positive cells for each case is reported on a scale from 0% to 100%. Then the mean value of five images was calculated.
